# BM-Net: CNN-Based MobileNet-V3 and Bilinear Structure for Breast Cancer Detection in Whole Slide Images

**DOI:** 10.3390/bioengineering9060261

**Published:** 2022-06-20

**Authors:** Jin Huang, Liye Mei, Mengping Long, Yiqiang Liu, Wei Sun, Xiaoxiao Li, Hui Shen, Fuling Zhou, Xiaolan Ruan, Du Wang, Shu Wang, Taobo Hu, Cheng Lei

**Affiliations:** 1 The Institute of Technological Sciences, Wuhan University, Wuhan 430072, China; jinhuang@whu.edu.cn (J.H.); liyemei@whu.edu.cn (L.M.); mplong@connect.ust.hk (M.L.); xiaoxiaoli@whu.edu.cn (X.L.); wangdu@whu.edu.cn (D.W.); 2 Department of Pathology, Peking University Cancer Hospital, Beijing 100142, China; victor.liu76@163.com (Y.L.); swsu8796@163.com (W.S.); 3 The Department of Hematology, Zhongnan Hospital, Wuhan University, Wuhan 430071, China; shenhui@znhospital.cn (H.S.); zhoufuling@whu.edu.cn (F.Z.); 4 Department of Hematology, Renmin Hospital, Wuhan University, Wuhan 430071, China; ruanxl0827@126.com; 5 Department of Breast Surgery, Peking University People’s Hospital, Beijing 100044, China; shuwang@pkuph.edu.cn

**Keywords:** lightweight, MobileNet-V3, bilinear structure, whole slide image, breast cancer detection

## Abstract

Breast cancer is one of the most common types of cancer and is the leading cause of cancer-related death. Diagnosis of breast cancer is based on the evaluation of pathology slides. In the era of digital pathology, these slides can be converted into digital whole slide images (WSIs) for further analysis. However, due to their sheer size, digital WSIs diagnoses are time consuming and challenging. In this study, we present a lightweight architecture that consists of a bilinear structure and MobileNet-V3 network, bilinear MobileNet-V3 (BM-Net), to analyze breast cancer WSIs. We utilized the WSI dataset from the ICIAR2018 Grand Challenge on Breast Cancer Histology Images (BACH) competition, which contains four classes: normal, benign, in situ carcinoma, and invasive carcinoma. We adopted data augmentation techniques to increase diversity and utilized focal loss to remove class imbalance. We achieved high performance, with 0.88 accuracy in patch classification and an average 0.71 score, which surpassed state-of-the-art models. Our BM-Net shows great potential in detecting cancer in WSIs and is a promising clinical tool.

## 1. Introduction

Breast cancer is the most common cancer in women [1] and poses a serious threat to women’s health all around the world. An essential step in managing breast cancer is WSI diagnosis, which provides guidelines for treatment [2]. Traditionally, pathologists evaluate hematoxylin and eosin (H&E) staining slides to generate a diagnosis and breast cancer grading result [3,4]. Due to the high spatial resolution, pathologists spend more time evaluating a whole slide image (WSI) than other medical images. Furthermore, there is a shortage of experienced pathologists, who require years of training and examination, which creates challenges for cancer centers [5]. In the automatic analysis of WSIs, glass slides are digitized to produce on-screen WSIs, and artificial intelligence, in particular deep learning technology, is applied [6,7,8]. The emergence of digital WSIs has made it possible to introduce deep learning. Nowadays, deep learning achieves better results than human expertise in many computer vision tasks [9,10,11], such as autonomous driving [12], bone age assessment [13], and endoscopic diagnosis [14]. With the benefits of time-saving and the use of fewer computational resources, deep learning has become a promising computer-aided diagnosis tool [15] and a trend in medicine [16]. Furthermore, histologists screen slides at maximum magnification and grade cancer by evaluating nucleus density, the mitosis process, etc., which takes approximately 30 min per WSI [17]. Thus, the requirement for experienced histologists and labor intensity have become restrictions for pathology diagnosis. To resolve these problems, deep learning is an option that reduces pathologist workloads, improves diagnosis performance, and saves time. In this paper, we adopted a deep learning algorithm to detect the suspect areas of breast histological slides by auxiliary diagnosis in grading cancer. By acquiring enormous slides to train our BM-Net so that the model robustness is sufficient for clinical settings, a BM-Net which performs as well as pathologists could liberate pathologists from heavy workloads and ensure cancer diagnosis by double-checking.

Currently, some researchers divide the whole slide into small patches and then train the network with patches, rather than directly utilizing the WSI [18]. Given the limitation of computational resources, state-of-the-art hardware is unable to calculate the gigapixel slide directly [19]. If the WSI is compressed into a minimum image, the cellular morphology will lose discriminative features for the diagnosis of breast cancer. There are two methods for locating suspicious regions in a WSI. The first method processes the patches that split from the WSI. Vidyarthi et al. [20] utilized LeNet-5 to train a binary classification. Araujo et al. [21] introduced the support vector machine classifier with a convolutional neural network (CNN), achieving an accuracy of 77.8% for four classes. Vesal and Ravikumar et al. [22] adopted Inception-V3 to train a four-class microscopy image dataset, achieving 97.08% accuracy. Similarly, Ferreira et al. [23] utilized the pretrained Inception-ResNet-V2. Anupama et al. [24] creatively adapted the capsule network to process patches and achieved improved performance. Huang et al. [25] applied the adapted Residue network while at the same time introducing deep feature fusion. In the end, this method achieved an accuracy of 98.5%. Senousy et al. [4] utilized an entropy-based elastic ensemble of deep convolutional neural networks to divide breast cancer into three invasiveness grades, achieving a grading accuracy of 96.15%. In summary, many deep learning engineers utilize patches as research objects. Because patches are so small and isolated causing the patches no spatial correlation and cancer distribution of WSI, patches cannot meet the clinical needs. Another approach splits the WSI into patches with a single label, then stitches together the results for each patch to produce a whole prediction map of cancer distribution. Ni et al. [26] applied a DeepLab architecture, which performed processing quickly and efficiently, but required an extensive dataset to train the network. Patil et al. [27] presented a modified U-Net based on an auto-encoder that achieved better performance than the DeepLab network in the binary task. Moreover, Das et al. [28] adopted a multi-instance network to exclude input disturbance and acquire cancer features. Kanavati et al. [7]. used the EfficientNet-B1 to detect breast invasive ductal carcinoma, with loose annotation. Finally, the network had universality and performed well in three surgical devices, but the model needed a lot of datasets to remove disturbances resulting from false positives. Therefore, deep learning networks have become a potential tool in pathology, especially detecting cancer regions.

With regard to breast cancer in WSIs, segmentation and classification networks for deep learning are used to detect abnormal regions. The segmentation method converts the detection task into a segmentation task. For instance, Galal et al. [29] used a candy cane architecture to segment the WSI on the basis of morphology. It was able to achieve a score of 0.45; however, the model was sensitive to subtle differences among cancers, resulting in a poor prediction map. Murata et al. [30] applied the U-Net to detect breast cancer. However, the method performed poorly and was unstable at the margins of the cancer regions. Li et al. [30] utilized DeepLab-V2 and achieved a score of 0.52. In summary, after examining these segmentation methods, we found that they performed poorly in the WSI detection task, because the WSIs lost important features when they were heavily compressed. The classification method converts tasks into classifications using images with particular labels. Because of their huge spatial dimensions, networks cannot infer WSIs directly. After splitting WSIs into patches, a network is trained to study target features by the corresponding label. Kohl et al. [31] utilized DenseNet-161 to distinguish each patch. However, DenseNet-161 required a much larger training dataset to acquire cancer features than former networks, resulting in poor performance because only 10 WSIs could be used in the BACH challenge. In the competition, we found that Li and Jia et al. [30] both used the ResNet network to detect breast cancer, achieving a 0.52 score. Marami and Ciga et al. [32,33] applied a series of ensemble networks and attention mechanisms, respectively. However, the best performance was achieved by Kwok [34] in the BACH competition, with scores of 0.6929 in detecting three breast cancers using a custom Inception-ResNet-V2 network. However, Kwok applied a heavyweight model that required a lot of time to train the parameters. Because differences between cancers are small, segmentation networks find it more challenging to distinguish cancer in WSI than classification networks. However, classification networks still face challenges in identifying the four classes of breast cancer than the binary task.

In this paper, we propose the bilinear MobileNet-V3 (BM-Net) for the detection of cancer regions in breast cancer WSIs. Due to the complexity and spatial dimensions of WSIs, we split the WSI into patches, then created a network to train the breast cancer detector. Firstly, we selected a lightweight BM-Net to extract breast cancer features, which combined MobileNet-V3 and the bilinear structure. In this way, BM-Net can save time and computational resources, and be easy to deploy the hardware. Moreover, the bilinear structure behaves well in fine-grained categorization classification, especially in detecting similar carcinomas such as in situ and invasive carcinomas. Secondly, we adopted a series of data augmentation techniques to improve diversity and dataset volume, increasing the network accuracy and specificity. These techniques were random flip, random rotation, random translation, random center-crop, and color jitter. Thirdly, because the numbers among classes were imbalanced, resulting in the tendency to predict the highest number of images as the largest number of classes, we introduced focal loss to balance the weights of the different numbers of four cancers. Fourthly, we stitched the WSIs, considering the neighboring patches, to solve the problem of overlapping samples, and reduced some erroneous predictions using majority voting in postprocessing. After these steps had been taken, BM-Net behaved stably and surpassed start-of-the-art networks in the field of breast cancer WSI detection. The main contributions of this work can be summarized as follows:

1. We used BM-Net to detect the ROI (region of interest) in breast cancer WSIs. The network was lightweight and stable because of its simple structure and small number of parameters.
2. We constructed an end-to-end network to process WSIs instead of a series of network cascades. This reduced computational resources and instability factors in the clinical setting.
3. We adopted the focal loss method to alleviate the imbalance between different classes. In the patch dataset, the number of invasive carcinoma patches was far larger than the others, therefore focal loss adjusted the model to study the remaining carcinomas.
4. For postprocessing, we applied majority voting to consider the effect of neighboring patches by analyzing prediction values from the spatial features.

## 2. Materials and Methods

### 2.1. Dataset Description

Our experimental dataset came from part B WSI segmentation of the BACH challenge [35]. The dataset consisted of 30 training WSIs for training networks and 10 test WSIs for match ranking. All WSIs are shown in RGB color mode after being stained using hematoxylin and eosin (H&E). The WSIs were scanned at 20× magnification, as pathologists focus on cell features and tissue morphology under maximum magnification. The dimensions of these WSIs are huge; for example, 54,721 × 46,305 pixels. In addition, only 10 WSIs of the training WSIs had ground truths; the remaining 20 WSIs were without annotations.

**Figure 1:**
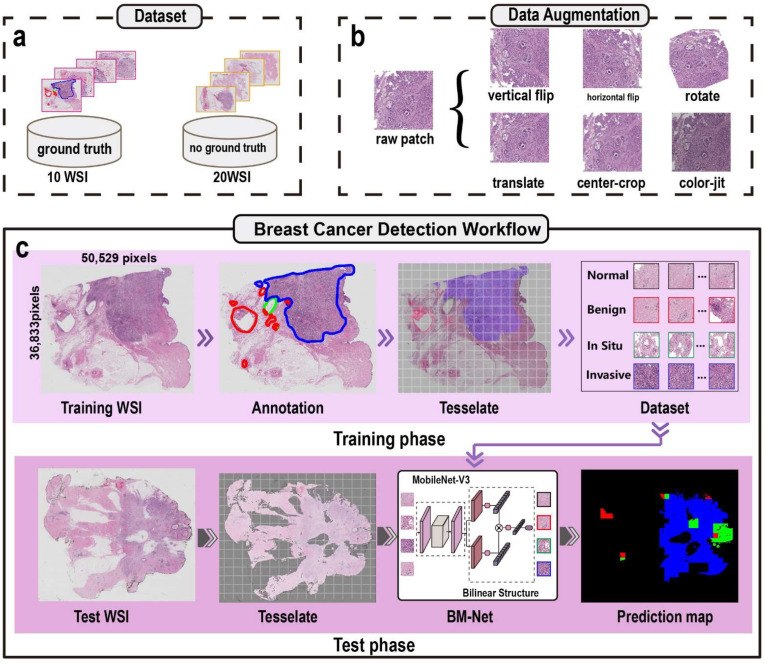
The entire breast cancer detection workflow. (**a**) The slide dataset includes 10 WSIs with and 20 WSIs without ground truth. (**b**) Data augmentation techniques, including horizontal and vertical flipping, translation, rotation, center-crop, and color jitter. (**c**) The breast cancer detection workflow consists of training and test phases.

We cooperated with histological experts from Peking University People’s Hospital and Peking University Cancer Hospital to annotate the 20 WSIs without ground truths, adding our dataset. Specifically, three pathologists labeled the remaining 20 WSIs using Aperial Image scope software. Firstly, two pathologists worked together to label all the WSIs. Next, a senior expert checked the results. If there were disagreements, we adopted the senior expert’s opinion as the final diagnosis. Finally, we acquired 30 WSIs to train the BM-Net. We discarded two slides (08, A01) because of discriminative differences from other breast cancer. We randomly allocated 23 WSIs for the training network and the remaining WSIs for evaluating the model’s performance, in a 4:1 ratio. Due to the gigapixel size of the WSIs and limitations of computational resources, the convolutional neural networks are unable to infer the WSIs directly. We split the WSIs into patches to match the network for overcoming appeal obstacles. In addition, we ensured that each patch included features, which are depicted in Figure 1.

### 2.2. Methods

The breast cancer detection workflow in Figure 1 consists of a training network phase and a test network phase. The former utilizes the patches with labels to train BM-Net to acquire breast cancer features, whereas the latter detects the suspect regions using the trained model. As shown in Figure 1, each phase consists of three modules: preprocessing, network, and postprocessing. Firstly, the preprocessing splits the WSI into small dimension patches to satisfy the computational capacity of the hardware. Secondly, the network, which is the most significant module in the breast cancer detection workflow, studies the cancer features and detects suspect regions in the WSI. Thus, the network directly affects the performance of detecting cancer. Thirdly, postprocessing stitches all the patches together and generates a prediction map to show the cancer distribution. Additionally, our breast cancer detection workflow is an end-to-end network, inputting an entire WSI to the BM-Net and directly providing a final prediction map.

#### 2.2.1. Preprocessing

We portioned the WSIs into small patches, which made it possible for the architecture to study the WSI dataset. This was necessary because WSIs are gigapixels in size and contain both breast tissue and background. However, as the background is not useful for the network, patches containing the background were discarded, which has the benefit of improving the training speed. For detail, we adopted the operational test support unit (OTSU) method to subtract tissue regions, then applied median blur and morphology operations to eliminate petty noise. Thus, we obtained a corresponding tissue mask for the subsequent sampling patches. Secondly, we acquired the patches by sliding the windows and saving eligible images where the ratio of tissue was greater than 0.5. Next, we assigned a label to each patch based on the ground truth. In addition, other cancer regions of the patches were filled with white color (255,255,255). In detail, we rescaled the WSIs at 12× magnification, then we cut patches in terms of the refined annotation mask. We applied the sliding windows technique, with dimensions of 2048 × 2048 pixels. Tessellating the WSIs, we performed window sliding in a stride of 1024 pixels, resulting in overlaid sampling and a dataset with more patches. Finally, we saved the center coordinates of each patch, which is essential for stitching together the whole image prediction map. With regard to the label, we assigned normal, benign, in situ carcinoma, and invasive carcinoma with labels 0, 1, 2, and 3, respectively. After conducting preprocessing, we obtained the training images dataset.

We applied data augmentation techniques to expand the patches dataset, improving the diversity of the dataset and network stability. These were horizontal flip, vertical flip, random rotation, random translation, random center crop, random color jitter, and random resizing of the patch. We selected one or more of the data augmentation techniques to generate new patches. We set augmentation technique parameters to avoid generating blank and distorted images. For rotation, patches needed to rotate at a small angle, 15 degrees, to avoid discarding too much information from the four corners. For translation, we chose 0.1× width and 0.1× height as the large scale, to ensure tissue was present in the patch. Next, because cellular features and morphology are crucial in cancer detection, patches were cropped on small scales, for example, 2000. Because of the color differences in staining WSIs, color jitter provided additional color expression to imitate the H&E staining variation, promoting the diversity of the image color. Thus, we set the brightness at 0.7, hue at 0.05, and saturation at 0.1 to keep the values reasonable. Generally, data augmentation techniques added useful images for BM-Net.

To train the network more effectively, we divided all of the images into training and validation datasets for our experiment. After conducting preprocessing, we acquired 21,540 normal, 3648 benign, 4672 in situ carcinoma, and 15,920 invasive carcinoma patches. All these labeled patches were randomly divided into the training and validation datasets at a ratio of 4:1. In particular, we ensured that training patches did not belong to the validation dataset. The test WSIs underwent the same preprocessing as the training WSIs. In addition, we saved the coordinates of all the patches for stitching the prediction map in the postprocessing stage.

#### 2.2.2. Network Architecture and Training

**Figure 2:**
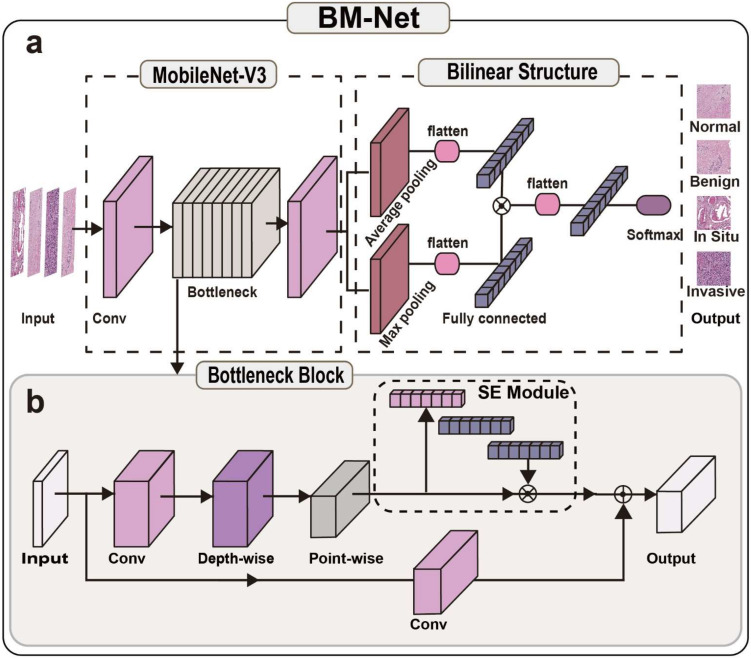
BM-Net. (**a**) BM-Net structure. The model consists of MobileNet-V3 and a bilinear structure. The bilinear structure replaces the classifier of the MobileNet-V3. (**b**) The bottleneck block structure consists of depthwise-separable convolution and a squeeze–excite (SE) module.

With the development of computer vision, many new deep learning techniques have been proposed for medical image classification tasks. In particular, the bilinear algorithm extracts distinguishing features and improves classification performance. Thus, we applied a bilinear structure to distinguish the four classes of breast tissue. To improve the trained model, we used MobileNet-V3 [36] as the backbone of our architecture. Firstly, MobileNet-V3 is a lightweight model that processes images quickly and efficiently. Secondly, the module architecture requires fewer computational resources, which makes it more suitable for use with the bilinear algorithm [37] because the fully connected layer is extremely time-consuming. As the architecture consists of the bilinear structure and MobileNet-V3, we called our model network BM-Net. The structure is depicted in Figure 2.

(1) The proposed method

We proposed the BM-Net to distinguish breast cancer in WSIs. To reduce the time required for analyzing the WSIs, we introduced a lightweight MobileNet-V3 to extract abnormal features. Additionally, because the differences between breast cancers are similar, accurate predictions depend on more valuable features. Thus, we replaced the classifier of MobileNet-V3 with the bilinear structure. In particular, we utilized the max pooling layer and the average pooling layer to extract more useful information, then we fused all information for the final prediction. Finally, we propose the BM-Net by applying the MobileNet-V3 and bilinear structure, with benefits in terms of time consumption, efficiency, and accuracy.

For the BM-Net, the head is MobileNet-V3 and the tail is the bilinear structure. Firstly, the model applies the standard convolution to add the dimension for sequential bottlenecks. Next, the network introduces batch normalization, which is used to normalize the values of the outputs and activation function, adding the non-linear capability to extract breast cancer features. Then, the module adopts a series of bottleneck blocks to extract discriminative features. Moreover, each bottleneck block contains a 1 × 1 convolution to improve the dimension of inputs for the linear activation function because it performs better at high dimensions. Sequentially, it uses depth-separable convolution to extract subtype tumor features. This conserves enormous computation resources, leading to time saving and efficiency. Additionally, each bottleneck adopts a squeeze–excite (SE) module and shortcut module to enhance the ability to focus on the essential regions. There are two types of bottleneck modules. In this paper, our experiment utilized the small version to acquire useful features. Hyperparameters of each bottleneck block are presented in detail in Table 1, which determined whether to adopt the SE block and shortcut with the ResNet structures. Finally, at the tail of the BM-Net, the module replaces the classifier of MobileNet-V3 with the bilinear structure to analyze information from the preview operation layers. In contrast with the original MobileNet-V3, we introduced the bilinear structure to make full use of patch features from the MobileNet structure [38]. In the training model phase, the focal loss [39] method was also essential to ensure the model performed stably.

(2) Bilinear structure

The bilinear structure [37] behaves well in fine-grained categorization classification because it keeps the translation invariance, and is good at classifying similar and complex images. This is particularly helpful for identifying the difference between in situ carcinoma and invasive carcinoma in histopathology microscopy images, which are complex and similar. Therefore, our task matched the application scenario of the bilinear structure. The bilinear structure extracts both the max features from the former layer and the average features, so the bilinear structure considers more information to infer the final prediction results.

The bilinear structure [37] was introduced into the breast cancer detection network, and calculated the max features and the average features. Firstly, we acquired two results of the input images by applying average pooling and max pooling. Next, the two results were merged in a fully connected layer to extract the essential features. These two outputs were multiplied with each other to form a conjunction of the two values. Using preview operations, the information from the pooling layers was fused. Finally, we acquired the last class label by carrying out a full connection operation. The detailed steps of the last phase can be seen in Figure 2.

(3) MobileNet-V3

Utilizing methods to decrease computational resources without compromising accuracy, MobileNet [40,41] requires neither excellent hardware nor excessive time. MobileNet consists of depthwise-separable convolution, an SE module, a linear bottleneck, and the Net-Adapt search application [29,33,34]. The depthwise-separable convolution structure is a key module to dramatically improve efficiency without sacrificing accuracy. However, compared to standard convolution, depthwise-separable convolution [40] divides standard convolution into depthwise convolution and pointwise convolution. Specifically, depthwise-separable convolution calculates the output by first multiplying each layer kernel with each input channel; then, the results are processed using a 1 × 1 convolution. Thus, by introducing depthwise-separable convolution, we can theoretically reduce computation costs by 8 to 9 times compared with standard convolution. The SE module adopts the attention map to help the network extract meaningful features, which greatly improves the accuracy of BM-Net.

The linear bottleneck module was created from MobileNet and makes full use of the activation function, which performs better at high dimensions. The linear bottleneck uses a non-linear activation function at the head because of the expanded dimension input, whereas it uses a linear activation function at the tail, at the low dimension. The result shows that each linear bottleneck block is sensitive to meaningful features and translates the computation vectors into the next layers.

MobileNet-V3 has become a well-known lightweight and efficient architecture in computer vision, and MobileNet_V3 can quickly infer cancer features. The streamlined model consists of three parts: standard convolution, a bottleneck module, and a final processing module. In the network, the first module is used to prepare for the bottleneck module, improving the dimensions only. The bottleneck module is the main structure, and uses various methods to extract meaningful features and the foundation of the architecture. In the final module, some fully connected layers are applied to select the important information and provide the final prediction result for the task.

(4) Focal loss function

(1) $$FL(p_{t})=-\alpha_{t}{\left(1-p_{t}\right)}^{\gamma }\log(p_{t})$$

The focal loss function addresses the class imbalance in training the BM-Net network to make sure the architecture studies four class features of breast tissue. In preprocessing, we split the WSIs into patches as input for BM-Net. Because the area of invasive carcinoma is far greater than that of benign and in situ carcinoma, the number of patches for the three carcinomas will vary greatly after preprocessing. Thus, the BM-Net tends to predict more patches as being invasive, neglecting benign and in situ carcinomas. To avoid the influence of class imbalance and to acquire useful features from benign and in situ carcinoma, we introduced the focal loss function to reduce the weight of loss value from the invasive carcinoma. In Formula (1), $\alpha_{t}$ and γ are hyperparameters for calibrating the weights of the BM-Net; moreover, the $p_{t}$ is the probability BM-Net performing a correct prediction.

### 2.3. Postprocessing

**Figure 3:**
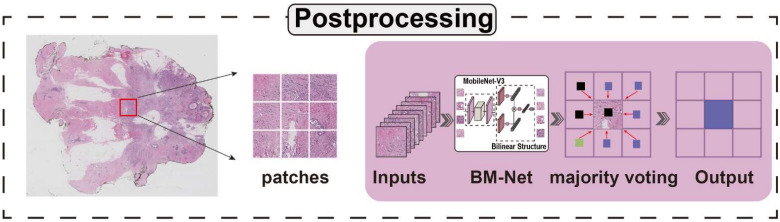
Postprocessing. Each patch prediction comes from the majority voting of neighboring patches.

As shown in Figure 3, postprocessing is essential for creating the final WSI prediction map. After analyzing patches from the whole slide image, we need to stitch these patches together to generate the WSI prediction map. During the test phase, we generated patches and saved their coordinates. When all of the patches were fed into the trained model, we obtained predictions for these patches. Combining the coordinates, predictions, and sizes of the patches, we stitched together the prediction map. In addition, we used overlap sampling, as overlapping regions are influenced by neighboring patches. To improve the selection of the value of the overlapping areas, we used majority voting, picking the most frequent values as the final label. The final prediction map also benefited from this method, because it considers the neighboring digital information, referred to spatial relations, which is essential in WSI. Compared with the directly stitched prediction map, majority voting performs better, especially in small suspect regions. We observed that the majority voting prediction map was more likely to resemble the ground truth. In addition, we utilized convolution conditional random fields (CRFs) to remove noise from the corresponding prediction map.

### 2.4. Evaluation Metric

The WSI prediction map was the final result of our task. We used a slide level evaluation metric to analyze our model performance [15]. In Formula (2), p is the predictive class (0, 1, 2, or 3), g is the ground truth class, i is the linear index of a pixel in the image, N is the total number of pixels in the image, and bin is the binarized value; for instance, the value is 0 if the label is 0 and 1 if the label is not 0. This $Q_{Score}$ is based on the accuracy metric, aiming at penalizing the predictions that are farther from the ground truth value. In other words, $Q_{Score}$ belongs to 0~1, and if the $Q_{Score}$ is close to 1, the map is similar to the ground truth, demonstrating the better performance of the model.

(2) $$Q_{Score}=1-\frac{\sum_{i=1}^{N}\left|p_{i}-g_{i}\right|}{\sum N_{i=1}\max(\left|g_{i}-0\right|,\left|g_{i}-3\right|)\times \left[1-(1-p_{i,bin})(1-g_{i,bin})\right]}$$

The $Q_{Score}$ is an evaluation metric for measuring the performance of each WSI. The $Q_{Score}$ penalizes regions where the prediction is farther away from the ground truth according to the arithmetical formula. We observed that the $Q_{Score}$ was influenced by the blank regions in annotation, which introduced some deviations. Although in the evaluation metric $Q_{Score}$ contains some shortcuts, $Q_{Score}$ was utilized by the BACH challenge to evaluate all the networks. Thus, other teams who used the breast cancer WSI dataset calculated $Q_{Score}$ in the results analysis section to evaluate the performance of the network [29,31,32,33,34].

### 2.5. Hyperparameter Setting

In the training stage, we applied a batch size of 40, a learning rate of 0.001, and 100 epochs to train our model. Considering the shape of our input images, we resized the patches to 1024 × 1024, reducing the computational resources. Moreover, we adopted the Adam solver [42] to optimize model parameters during the training phase. All the experiments were conducted in Pytorch [43] under an Ubuntu OS cloud server with an Intel Xeon(R) CPU E5-2680 v4 @2.40 GHz, 40 GB of RAM, and an NVIDIA Tesla P40 GPU with 24 GB of memory.

## 3. Results

After conducting the experiments, we analyzed the performance and the consumption time of BM-Net during breast cancer detection. To better evaluate the performance, we adopted $Q_{Score}$ to analyze the results. Additionally, we compared the performance of majority voting and direct stitch as postprocessing to see whether or not there was any improvement in the generation of the prediction map.

### 3.1. Ablation Experiment

**Figure 4:**
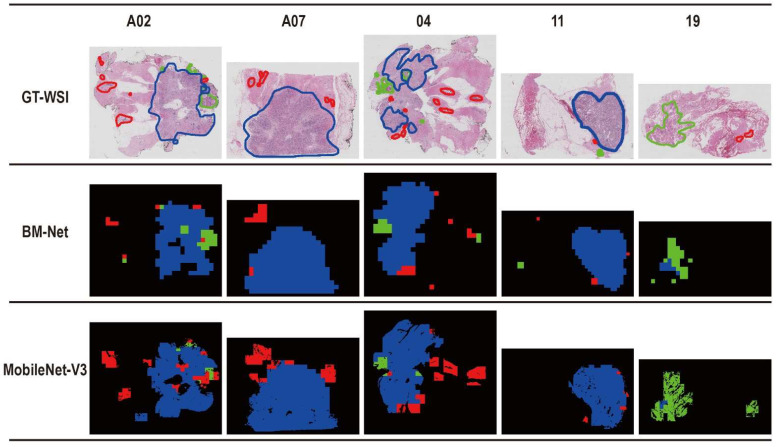
Ablation experiment. Performance of BM-Net and MobileNet-V3. GT-WSI denotes the ground truth of the WSI. The red, green, and blue areas or the lines regions denotes benign, in situ carcinoma and invasive carcinoma, respectively.

We used BM-Net to identify the abnormal regions in the breast WSI, and therefore we conducted a pair of ablation experiments to prove the effectiveness of the bilinear structure. BM-Net consists of MobileNet-V3 and the bilinear structure, replacing the classifier of MobileNet-V3 with the bilinear structure. In this experiment, we utilized the same dataset, preprocessing method, hyperparameters, and postprocessing techniques. The dimensions of input patches were 1024 × 1024 pixels. We added an average pooling layer before the classifier of MobileNet-V3 because the size of the input image was larger. After the experiment, we compared the evaluation metric $Q_{Score}$ in Table 2 and the prediction map in Figure 4. Comparing the value of each test slide, we found that BM-Net performed better in A02, A07, and 19 slides, and it improved the average $Q_{Score}$. In Figure 4, we found that the prediction map of BM-Net was more likely to the annotation whereas MobileNet-V3 tended to predict more cancer regions. The results of the ablation experiment show that the bilinear structure improves the accuracy of the network.

**Figure 5:**
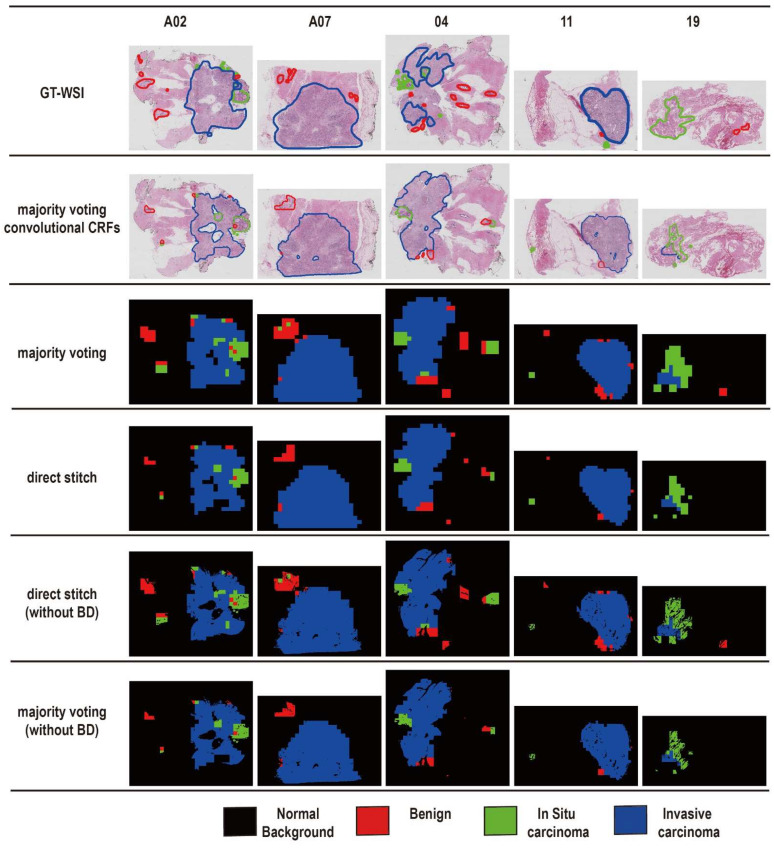
Postprocessing performance. GT-WSI denotes annotation. Direct stitch denotes stitching the prediction map directly. Majority voting convolution CRFs (conditional random fields) denotes that we generate a prediction map using voting. BD means background. The red, green, and blue areas or the lines regions denotes benign, in situ carcinoma and invasive carcinoma, respectively.

In our experiments, BM-Net performed efficiently and saved time. Because we used a lightweight network BM-Net to process the whole slide, which is fast and efficient compared with other deep learning architectures, predicting a whole slide image took about 1.3 min. Furthermore, the preprocessing and postprocessing took approximately 2 min, respectively. Therefore, 3.5 min is sufficient to predict a whole slide image. BM-Net both infers efficiently and generates better performance, which is shown in Figure 5.

### 3.2. Performance

In our paper, we converted WSI suspect region detection into a four classes classification task and then generated a prediction map to show cancer distribution. In this section, we set out the performance of BM-Net and also show the differences between postprocessing methods. We also evaluate the performance using qualitative and quantitative analyses. Specifically, we tested five WSIs and generated the final prediction maps. To clearly show the results, all the prediction maps are displayed in Figure 5. GT-WSI means the ground truth of the WSI. Four postprocessing methods are shown. Direct stitch generated the prediction map by stitching patches directly. Majority voting convolution CRFs (conditional random fields) means that we utilized convolution CRFs to optimize the prediction map by eliminating the output noise. Majority voting generated the prediction map by voting from neighboring classes. Direct stitch (without BD) means that the prediction map was generated by stitching it directly and removing the blank area. Majority voting (without BD) removed the blank area of the majority voting map.

The results are shown in Figure 5. According to the performance of the five WSIs, we found that the invasive carcinoma regions were mainly detected by BM-Net. In particular, comparing the pictures of the ground truth and the direct stitch, we observed that the colored region of the two pictures was similar, indicating that BM-Net could detect the cancers. We also found that the breast cancer detector was more accurate in inspecting the invasive carcinoma and neglected the normal tissue regions. Additionally, we found that BM-Net was sensitive to dark-purple-colored regions. Differences between the two postprocessing methods showed better accuracy by neglecting noise predictions in the large area of one class. In particular, the performance of the majority voting method is more effective than the other methods, because majority voting utilizes more information from neighboring patches. We also evaluated the background (BD) effect. Because of the existence of background in the annotation, the final output would be disturbed if all prediction labels were directly stitched using 2048 × 2048. In the end, we excluded the background using a tissue mask, which was generated by the OTSU algorithm. After discarding the suspect regions of background, the edges and pores of breast tissue were more accurate. Finally, comparing all images, we concluded that majority voting without background delivered better performance than the other techniques.

### 3.3. Quantitative Evaluation

We represent BM-Net performance using the evaluation metric $Q_{Score}$. Table 3 displays the detailed values for each slide and each technique. Additionally, Table 3 corresponds to Figure 5, where each value was calculated using the corresponding prediction map. When we calculated the results of the prediction map without background, we adjusted g by excluding the background in the annotation region. The evaluation metrics $Q_{Score}$ without background, whether using majority voting or direct stitch postprocessing, all performed better than those with background, by about 4 percentage points. By comparing the metric values, we found that majority voting values were slightly lower than those for the direct stitch method.

We found that majority voting performed well in the A07, 04, and 11 slides. With regard to A02 and 19, we observed that the ability of majority voting decreased when the slide contained more blank in tissue regions. On the one hand, majority voting performed well in most of the test WSIs, and on the other hand, the prediction map created by the majority voting method presented a more accurate result in qualitative analysis.

### 3.4. Comparison with Existing Methods

We observed that our structure performed better than the other methods shown in Table 4. These architectures applied the same breast cancer WSI dataset and evaluation metric as ours. They applied either segmentation networks or classification networks. Galal et al. [29] utilized a candy cane network to detect breast cancer, which performed poorly in terms of the ROI. Murata et al. [30] applied U-Net to detect breast cancer, which performed better than candy cane method. The best performance in terms of segmentation was DeepLab-V2, which achieved a 0.52 score. However, classification networks perform better than segmentation networks in detecting cancer in WSI. DenseNet and ResNet perform worse than segmentation, but the other methods all perform better. Jia et al. [30] applied the ResNet-50 network to classify each patch and this performed to the same level as DeepLab-V2. Marami et al. [32] created an ensemble network that consisted of Inception-V3 and ResNet34, which achieved scores of 0.553. The best performance in processing breast cancer WSIs was demonstrated by an Inception-ResNet-V2 network proposed by Kwok [34], which won first place in the BACH challenge. Our BM-Net consists of MobileNet-V3 and the bilinear structure, after calculating the evaluating metric of 0.71. In addition, we also calculated the number of parameters (NMP), and the floating-point operations (FLOPs). In Table 4, we calculated NMP from the open-access code, but some networks that are not online were not included. Comparing the NMP, we found that BM-Net has fewer parameters than other networks. Using fewer parameters results in less inferring time, thus, BM-Net is a lightweight network. The FLOPs metrics indicate the complexity of the model, and the BM-Net had the fewest FLOPs among these networks. Therefore, BM-Net was able to process breast cancer images quickly. By analyzing Table 4, we found that BM-Net outperformed other networks in the WSI suspect region detection task. Our proposed architecture, BM-Net, is the best at detecting breast cancer regions because the bilinear structure is good at distinguishing similar images, such as in situ carcinoma. Furthermore, BM-Net is able to quickly acquire prediction results because it is a lightweight classification network with fewer parameters and FLOPs.

## 4. Discussion

Our proposed BM-Net architecture consists of MobileNet-V3 and a bilinear structure, achieving a better performance in detecting abnormal regions in breast cancer WSIs. Making full use of the lightweight MobileNet-V3 and the bilinear structure, which has good discriminatory ability, BM-Net inferred benign, in situ, and invasive carcinoma quickly and accurately. In this paper, we also used data augmentation techniques, focal loss function, and majority voting during the experiment, helping BM-Net to study cancer features. In addition, we conducted an ablation experiment and compared performance and computational resources. BM-Net worked better than MobileNet-V3, indicating that the bilinear structure distinguishes breast cancers. Furthermore, its NMP and FLOPs are far fewer than those for other networks, demonstrating that BM-Net consumes less computational resources. In the test phase, we observed that the prediction maps showed abnormal ROIs, especially in detecting invasive carcinoma. In summary, BM-Net was efficient and better at detecting breast cancer.

The abnormal regions could be screened out by our breast cancer detection workflow. Thus, the network aided the pathologist in evaluating the WSI. When BM-Net is introduced into the pathology field in clinical settings, it will benefit pathologists and hospitals. Firstly, BM-Net can reduce the checking time significantly, from 30 min to 3.5 min per WSI. Secondly, it can improve objectivity without the influence of experience and pathologists. Thirdly, it can assist in relieving the shortage of pathologists in some cancer centers. In the future, pathologists will benefit from BM-Net.

Deep learning is a promising tool in the pathology field, but there are still difficulties in screening the abnormal regions in complex WSIs. The differences between cancer subtypes are subtle, and sometimes, senior experts make mistakes when distinguishing similar regions. Firstly, hardware restrictions limit the size of images, so the network is unable to process the WSI directly. Secondly, if the WSIs are cut into patches to match the network limitations, we lose the spatial information around each patch. In the future, deep learning engineers will focus both on creating more efficient networks and optimizing the breast cancer detection workflow.

## 5. Conclusions

In this paper, we presented a lightweight BM-Net to detect cancer regions in WSIs. We obtained better performance in detecting the tumor regions of WSIs using our workflow. BM-Net consists of a bilinear structure and a lightweight MobileNet-V3. Thus, BM-Net can acquire valuable features using the bilinear structure and process efficiently. During the training phase, we augmented the patch dataset by overlap sampling and a variety of image augmentation techniques. After training the network, we tested the WSIs and obtained better performance using the majority voting and convolution conditional random fields methods as postprocessing. Finally, the network analyzed one WSI within 3.5 min, demonstrating its potential for utilization in the clinical setting.

Our BM-Net performs well in BACH breast cancer datasets. However, due to the difficulty in obtaining clinical data, our well-trained BM-Net may fail to meet situations where the WSIs exist with variability and difference during production. In future research, we intend to collect annotated clinical data and design a generalized, fully automatic system to detect breast cancer in WSI.

## Figures and Tables

**Table 1:** MobileNet-V3 small parameters of BM-Net.

Operator Type	Kernel Size	Expand	Out	Stride	SE	NL
Conv2d	3 × 3	-	16	2	-	h-swish
Bottleneck	3 × 3	16	16	2	√	ReLU
Bottleneck	3 × 3	72	24	2	-	ReLU
Bottleneck	3 × 3	88	40	1	-	ReLU
Bottleneck	5 × 5	96	40	2	√	h-swish
Bottleneck	5 × 5	240	40	1	√	h-swish
Bottleneck	5 × 5	240	40	1	√	h-swish
Bottleneck	5 × 5	120	48	1	√	h-swish
Bottleneck	5 × 5	144	48	1	√	h-swish
Bottleneck	5 × 5	288	96	2	√	h-swish
Bottleneck	5 × 5	576	96	1	√	h-swish
Bottleneck	5 × 5	576	96	1	√	h-swish
Conv2d	1 × 1	-	576	1	√	h-swish

Expand denotes the expanded number of convolutional filters. SE denotes the squeeze–excite module. NL denotes the non-linearity function. BN denotes batch normalization. √ denotes application of squeeze–excite module. While - denotes not application.

**Table 2:** $Q_{Score}$ of the BM-Net and MobileNet-V3.

Slide	A02	A07	04	11	19	Average
MobileNet-V3	0.6737	0.8515	0.5349	0.8549	0.3911	0.6612
BM-Net	0.7264	0.8959	0.4826	0.8092	0.4375	0.6703

**Table 3:** Performance of the test dataset.

Slide	Majority Voting	Direct Stitch	Majority Voting (Without BD)	Direct Stitch (Without BD)
A02	0.7264	0.7681	0.7876	0.8358
A07	0.8959	0.8567	0.9273	0.8878
04	0.4826	0.4543	0.5242	0.4917
11	0.8092	0.7645	0.8498	0.8027
19	0.4375	0.6359	0.4453	0.6791
average	0.6703	0.6959	0.7068	0.7394

Direct stitch generated the prediction map by stitching patches directly. Majority voting generated the prediction map using voting. Without BD generated the prediction map without blank regions. The best two results are highlighted in red and blue, respectively.

**Table 4:** Quantitative results of various methods.

Team	Network	Average $Q_{Score}$	NMP (M)	FLOPs(G)
Galal et al. [29]	Candy Cane	0.45	-	-
Kohl et al. [31]	DeseNet-161	0.42	28.68	3.99
Vu et al. [44]	DenseNet, SENet, ResNet	0.495	7.98	22.74
Galal and Sanchez-Freire [30]	DenseNet	0.50	-	-
Murata et al. [30]	U-Net	0.50	31.04	54.76
Li et al. [30]	VGG16, DeepLab-V2 ResNet-50	0.52	25.56	21.53
Jia et al. [30]	ResNet-50	0.52	25.56	2153
Marami et al. [32]	Ensemble Network (Inception-V3, ResNet-34)	0.553	-	-
Ozan Ciga et al. [33]	SE-ResNet-50, L-DANN module	0.68	-	-
Kwok [34]	Inception-ResNet-V2	0.69	-	-
**BM-Net**	**MobileNet-V3,** **Bilinear module**	**0.71**	**2.56**	**1.27**

NMP: the number of parameters. FLOPs: floating-point operations. Bold means the best results.

## Data Availability

Publicly available datasets were analyzed in this study. These data can be found here: https://iciar2018-challenge.grand-challenge.org/Dataset (accessed on 24 April 2022).
